# Functional Analysis of the FZF1 Genes of *Saccharomyces uvarum*

**DOI:** 10.3389/fmicb.2018.00096

**Published:** 2018-02-06

**Authors:** Xiaozhen Liu, Xiaoping Liu, Zhiming Zhang, Ming Sang, Xiaodong Sun, Chengzhong He, Peiyao Xin, Hanyao Zhang

**Affiliations:** ^1^Key Laboratory for Forest Resources Conservation and Utilization in the Southwest Mountains of China, Ministry of Education, Southwest Forestry University, Kunming, China; ^2^Key Laboratory of Biodiversity Conservation in Southwest China, State Forest Administration, Southwest Forestry University, Kunming, China; ^3^College of Life Science, Jinggangshan University, Ji'an, China; ^4^Central Laboratory of Xiangyang No.1 Hospital, College of Basic Medical Sciences, Hubei Key Laboratory of Wudang Local Chinese Medicine Research, Hubei University of Medicine, Shiyan, China

**Keywords:** *S. uvarum*, *FZF1* gene, sulfite tolerance, functional analysis, qRT-PCR, transcriptome analysis

## Abstract

Being a sister species of *Saccharomyces cerevisiae, Saccharomyces uvarum* shows great potential regarding the future of the wine industry. The sulfite tolerance of most *S. uvarum* strains is poor, however. This is a major flaw that limits its utility in the wine industry. In *S. cerevisiae, FZF1* plays a positive role in the transcription of *SSU1*, which encodes a sulfite efflux transport protein that is critical for sulfite tolerance. Although *FZF1* has previously been shown to play a role in sulfite tolerance in *S. uvarum*, there is little information about its action mechanism. To assess the function of *FZF1*, two over-expression vectors that contained different *FZF1* genes, and one *FZF1* silencing vector, were constructed and introduced into a sulfite-tolerant *S. uvarum* strain using electroporation. In addition, an *FZF1*-deletion strain was constructed. Both of the *FZF1-*over-expressing strains showed an elevated tolerance to sulfite, and the *FZF1*-deletion strain showed the opposite effect. Repression of *FZF1* transcription failed, however, presumably due to the lack of alleles of *DCR1* and *AGO*. The qRT-PCR analysis was used to examine changes in transcription in the strains. Surprisingly, neither over-expressing strain promoted *SSU1* transcription, although *MET4* and *HAL4* transcripts significantly increased in both sulfite-tolerance increased strains. We conclude that *FZF1* plays a different role in the sulfite tolerance of *S. uvarum* compared to its role in *S. cerevisiae*.

## Introduction

*Saccharomyces uvarum* is a sister species of *Saccharomyces cerevisiae* that was initially considered a synonym of *Saccharomyces bayanus*, but is now considered a species in its own right. Subsequently, *S. uvarum* is an object of interest for scientists working on applied and fundamental research (Naumov et al., 2011; Masneuf-Pomarede et al., 2016). It hybridizes with its sister species, including *S. pastorianus, S. eubayanus*, and *S. cerevisiae*, to form hybrids that are important in the beer industry (Nguyen et al., 2011). As *S. uvarum* and *S. cerevisiae* both belong to *Saccharomyces* sensu *stricto*, these two species have similar characteristics, but in the later stage of the Sauvignon Blanc fermentation process, *S. uvarum* plays a more important role in producing alcohol from anaerobic respiration (Sipiczki and Ciani, 2002; Naumov et al., 2011). It has already been used in wine, but it is mainly used in cider (González Flores et al., 2017). It may have great potential in the wine industry in the future, however, as it can ferment at low temperatures (Zhang et al., 2015).

Yeast resistance to sulfite is an important technological characteristic for wine making (Nadai et al., 2016). In wine production, a variety of antioxidants are added, both to prevent oxidation and to inhibit the growth of unwanted yeast and bacteria. Sulfite, added as metabisulfite or as sulfur dioxide, is widely used in wine fermentation (Taylor et al., 1986), and usually at a dosage of 50 mg/L free sulfite in grape juice (Doneche, 1993). High concentrations of sulfite are also toxic to *Saccharomyces* cells, however (Nardi et al., 2010; Divol et al., 2012; Nadai et al., 2016). Hence, sulfite resistance has become one of the most important indexes for the screening of wine-making yeast strains.

In *S. cerevisiae*, the *SSU1* gene is the key regulator of sulfite tolerance. It acts as a “sulfite efflux pump” in cells, removing toxic sulfite from them (Donalies and Stahl, 2002). Meanwhile, sulfite also forms special flavors of wine, i.e., thiol esters, in the process, thereby improving the quality of the wine (Avram and Bakalinsky, 1997; Park and Bakalinsky, 2000; Donalies and Stahl, 2002; Nardi et al., 2010). Among wine yeast strains, two translocations have been identified that provide up-regulation of *SSU1* and increased sulfite tolerance (Pérez-Ortín et al., 2002; Yuasa et al., 2005; Zimmer et al., 2014). A second locus, *FZF1*, also provides sulfite resistance in *S. cerevisiae* (Breitwieser et al., 1993). *S. cerevisiae FZF1* is a transcription factor that has 203 targets and plays a positive role in the transcription of *SSU1* according to the yeast search for transcriptional regulators and consensus tracking (YEASTRACT) database (Teixeira et al., 2014). Efflux of sulfite via active transport appears to be the predominant method utilized by *S. cerevisiae* to improve the sulfite tolerance of the cell (Zimmer et al., 2014). Genome-wide microarray analysis of ribonucleic acid (RNA) expression identified a second possible transcriptional target for the *FZF1* gene besides *SSU1* in *S. cerevisiae*: *GCV1* (Hu et al., 2007), which encodes a T subunit of the mitochondrial glycine decarboxylase complex (McNeil et al., 1997; Piper et al., 2000).

In *S. uvarum*, relatively little is known about sulfite tolerance. Some strains show tolerance, however, and in one case, tolerance was shown to be linked to an allele of *FZF1* that had been introgressed from *S. eubayanus*, but was not linked to the *SSU1* locus (Zhang et al., 2015).

Studying the function of the *FZF1* gene in sulfite tolerance is vital for interpreting the metabolism mechanism of sulfite in *S. uvarum*. Here, we describe the construction of two different *FZF1*-over-expressing strains, one *FZF1*-silencing strain, and one *FZF1-*deletion strain. The function of the *FZF1* gene of *S. uvarum* was analyzed via sulfite-tolerant phenotype screening, polymerase chain reaction (PCR) analysis, growth curves, quantitative reverse transcription polymerase chain reaction (qRT-PCR), and transcriptome analysis.

## Materials and methods

### Materials

*S. uvarum* strain A9 (Zhang et al., 2015), pSilent, pYIP5, and *Escherichia coli* DH5α, were obtained from collections at Southwest Forestry University. Two RNA interference (RNAi) fragments, TTCATAAGACCATGTCATTTAA and TTAAATGACATGGTCTTATGAA, were designed using the website http://www.broadinstitute.org/rnai/public/seq/search, and were synthesized and constructed in a pSilent vector by Zoonbio Biotechnology Co. Ltd., China. The sequence of *FZF1-u* (Genbank accession number KY905342) was identical to that in the sulfite-tolerant *S. uvarum* strain A9, which originally came from *S. eubayanus* (Zhang et al., 2015), and the sequence of *FZF1-e* (Genbank accession number KY905343) was derived from *S. uvarum* strain ACY338. Fifteen percent of nucleotide sequences were different between *FZF1-u* and *FZF1-e*. *FZF1-u* and *FZF1-e* genes were synthesized with extra *BamH* I and *Sal* I sites at their termini, and constructed in the pGEM–T easy vector by Nextomics Biosciences Co. Ltd. The genotypes of all the strains used or created are listed in **Table 2**.

### Construction of the *FZF1* gene expression vector and transformation of *S. uvarum*

Procedures for the manipulation of plasmid deoxyribonucleic acid (DNA), and transformation and sequence validation, were performed as previously described (Sambrook et al., 1989). Using *BamH* I and *Sal* I, the PCR products of the *FZF1-u* and *FZF1-e* genes from the vectors and expression plasmid pYIP5 were digested. The digested fragments of the expression vector and PCR products were then ligated, and shuttle vectors were constructed. The vectors were again subjected to restriction analysis using *BamH* I and *Sal* I to confirm that the target gene had been correctly inserted into the vector. The vectors were also sent to Sangon (Shanghai Sangon Co., Shanghai, China) for sequencing to test for putative mutations. pYIP5-FZF1-u, pYIP5-FZF1-e, and pSilent-FZF1-u-s were transferred into *Escherichia coli* DH5α and the sulfite-tolerant *S. uvarum* strain A9 via electrotransformation as described as Kozak et al. (2014). DNA was extracted using the cetyltrimethyl ammonium bromide (CTAB) method described by Zhang et al. (2010). The *URA3* gene was used as a selectable marker for pYIP5-FZF1-u and pYIP5-FZF1-e transformants, and the hygromycin resistance gene was utilized for pSilent-FZF1-u-s transformants.

Candidate transformant colonies were subjected to PCR analysis to confirm. The primers used in the PCR are listed in **Table 2**. For the *FZF1* over-expression candidates, 10 transformant colonies of both *FZF1-u* and *FZF1-e* that could grow well on the medium containing 5-fluoroorotic acid (5-FOA) were selected for PCR analysis. The colonies of transformations could produce a 312-bp band specific to the *AmpR* gene. For the *FZF1* repression expression candidates, 10 transformed candidates with hygromycin resistance were selected for the PCR analysis. The transformant colonies were expected to generate a 760-bp specific band from the hygromycin resistance gene, and a 312-bp band specific to the *AmpR* gene.

### Construction of the *FZF1*-deletion strain

The methods used were the same as on the website (http://www-sequence.stanford.edu/group/yeast_deletion_project/deletions3.html). Candidate *FZF1-*deletion colonies used the *KanMX* gene as a selectable marker to screen. The deletion of the target gene was confirmed by PCR using the primer pairs FZF1-u-d-L1 _c_/FZF1-u-d-R1 _c_ and FZF1-u- d-L2 _c_/FZF1-u- d-R2 _c_ (see **Table 2**).

### Sulfite tolerance genotyping

After culturing for 24 h in liquid yeast extract peptone dextrose (YPD), 100 μL of the yeast colonies were diluted with liquid YPD (1:10, 1:100, 1:1000, and 1:10000). After dilution, 3 μL of the yeast colonies after dilution were inoculated onto fresh YPD plates containing 5, 10, 20, 40, 60, 80, 100, and 120 mM of sodium sulfite, and 80 mM of succinic acid (pH 3.5). After growing on the medium for 3 days at 30°C, the sulfite tolerance levels of the colonies were recorded by visual analysis of growth.

### RNA extraction and complementary deoxyribonucleic acid (cDNA) synthesis

Yeast colonies were collected after culturing for 24 h in liquid YPD without adding sulfite, then placed for 20 mins in liquid YPD with 20 mM of sodium sulfite and 80 mM of succinic acid, for RNA extraction. Total RNA was extracted using a Qiagen kit. The RNA was then reverse-transcribed into cDNA using a Fermentas kit.

### qRT-PCR analysis

qRT-PCR was performed according to the method described by Chen et al. (2008) with the ABI7500 fluorescence quantitative PCR instrument. Primers were FZF1-L_q_ and FZF1-R_q_ (see Table 1), and the 2^−ΔΔ*CT*^ method was used to assay the data (Livak and Schmittgen, 2001), with *PDA1* and *ACT1* as housekeeping genes for normalizing (Divol et al., 2006). Three parallel experiments were conducted for each gene.

**Table 1:** List of PCR primers.

**Gene**	**Sequence (5′ 3′)**	**Gene**	**Sequence (5′ 3′)**
FZF1-u-L	GCAGGATCCATGGCCAATACAAAGAAACCT	FZF1-e-L	5′-GCA*GGATCC*ATGGCAAATAAAAAGAAACTG
FZF1-u-R	CAGGTCGACTTAGTATTCAAATAA GCTCCT	FZF1-e-R	5′-CAG*GTCGAC*TTAGTATTCGAATAAGCTCCT
AMPR-L	GCTGCGCCTTATCCGGTAAC	HypR-L	CGTAGAAGCGCCGGAGATAG
AMPR-R	TCTGCGCGTAATCTGCTGCT	HypR-R	TACGCGTTCTTCCGGATCTC
KanMX-L	CGTACGCTGCAGGTCGAC	FZF1-u- d-L1	CCTTCGAGTCCACTCAATCC
KanMX-R	ATCGATGAATTCGAGCTCG	FZF1-u-d-R1	CTTCAGGTGGCAAAGAAAGC
FZF1-u-d-L2_c_	CACGAAGGCAATGAGTTGAA	FZF1-L_q_	CACGAAGGCAATGAGTTGAA
FZF1-u-d-R2 _c_	CTTCTTGCTGCTCTGCCTCT	FZF1-R_q_	CTTCAGGTGGCAAAGAAAGC
SSU1-L1_q_	5′-AAGCGGTGGGACATTTACAA-3′	ACT1-L_q_	CTGGGAYGAYATGGA RAAGAT
SSU1-R1_q_	5′-TGACCAGCAAACGCAAATAC-3′	ACT2-R_q_	GYTCRGCCAGGATCTTCAT
FZF1-u-d-L	TAAGAGAGCTACCGAGTGCTGGAACCATTTTTTGCTCAGGAGATGCGTACGCTGCAGGTCGAC	PDA1-L_q_	GGTCAGGAGGCCATTGCTGT
FZF1-u-d-R	TGAGAATGAATTGTACCCCACTTTTTTGACCAAAGGAGTCACTCGATCGATGAATTCGAGCTCG	PDA1-R_q_	GACCAGCAATTGGATCGTTCTTGG

*Underlining indicates BamH I and Sal I restriction sites; c, Used for confirming the deletion of the target gene; q, Used for RT-PCR*.

### Determination of growth curves of recombinant strains

Cultures of individual clones were grown for 64 h in liquid YPD, then diluted 100-fold into 0.05 L of fresh liquid YPD. The strains were the starting A9 strain, and transformed derivatives A9-FZF1-u, A9-FZF1-e, and A9-FZF1-u-s. Cultures were incubated at 30 degrees C, with shaking at 220 rpm, and sampled every 4 h, with growth measured using light absorption at 600 nm. Using the YPD liquid culture medium without inoculation as a control, the growth curves of the strains were plotted as optical density (OD600) against time. Different stages of the growth of the strains were used to calculate the maximum growth rates: For A9, A9-FZF1-u-s, and A9-FZF1-u-d we used the stage of 28 h to 32 h after inoculation; while for A9-FZF1-u and A9-FZF1-e, we used the stage of 20 h to 24 h. These stages were used as these were the points when the strains grew the fastest.

### Transcriptome analysis

For comparison of differential transcription genes between strains, next generation sequencing was employed to assess the RNA samples that were extracted from colonies cultured in YPD for 24 h, and then in 20 mM of sodium sulfite and 80 mM of succinic acid for 10 min. The RNA was prepared using Illumina TruSeq RNA-Seq kits, and RNA-Seq was performed by Nextomics Biosciences Co. Ltd using Illumina HiSeq™. The RNA-Seq data were submitted to the Genome Sequence Archive of Beijing Institute of Genomics (BIG) Data Center (publicly accessible at http://gsa.big.ac.cn, Accession No. PRJCA000414). Transcriptome analysis was performed using DEGseq (Wang et al., 2010) and DESeq (Anders and Huber, 2010).

### Statistical analysis

Data were analyzed by the graphing of histograms in Excel 2007 (Microsoft, Redmond, WA), mean ± standard errors (SE) were calculated, and significant differences between different strains were calculated using a one-way analysis of variance (ANOVA). Differences were considered statistically significant if the *p-*values were <0.05. For the transcriptome analysis, the *Q*-values were calculated based on *p* values to identify differentially expressed genes (Wang et al., 2010). If the *Q*-values were <0.01, differences were considered statistically significant.

## Results

### Sulfite resistance of transformations

**Figure 1:**
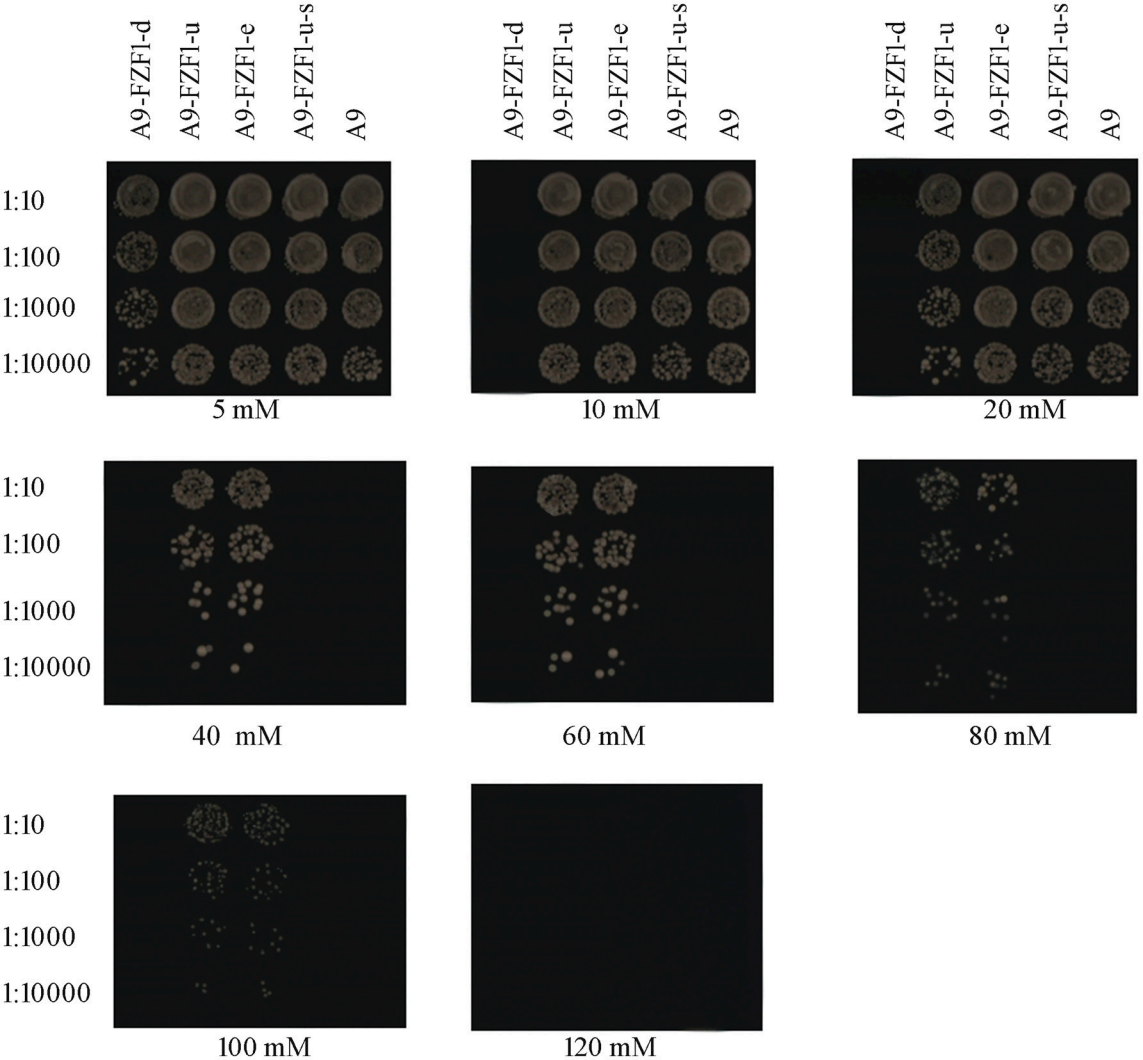
Drop off test experiment: yeast colonies were cultured for 24 h in liquid YPD without adding sulfite, then 100 uL of them were diluted with liquid YPD into 1:10, 1:100, 1:1000, and 1:10000 time. Three microliter droplets of yeast dilutions were inoculated onto fresh YPD plates containing 5, 10, 20, 40, 60, 80, 100, and 120 mM of sodium sulfite, and 80 mM of succinic acid (pH 3.5). After growing on the medium for 3 days, the sulfite tolerance levels of the colonies were recorded by visual analysis of growth.

Forty transformants (10 transformants for each strain) with the right genotypes (see Table 2) and PCR products were used for the sulfite resistant test. These 40 transformants, together with the starting strain A9, were inoculated and grown on YPD plates with different sulfite concentrations to screen the sulfite tolerant genotype. The results showed that A9-*FZF1-u* and A9-*FZF1-e* were the most tolerant colonies, and that they could grow on media containing 100 mM sulfite (see Figure 1). The sulfite tolerance of *FZF1-u-s* transformations, however, was not changed at all compared to the parent A9 strain as both could grow on 20 mM (see Table 1). These data demonstrate that over-expression of the *FZF1* genes from both *S. eubayanus* and *S. uvarum* could increase the sulfite resistance of *S. uvarum*, while the silencing of the *FZF1* gene via RNAi had no effect. The deletion strain could not grow on YPD with 10 mM sodium sulfite, but could grow on 5 mM. This indicated that the deletion of the *FZF1* gene in the *S. uvarum* genome may havedecreased the sulfite resistance.

**Table 2:** The genotype and sulfite resistance ability of different *S. uvarum* stains or transformations.

**Strains**	**HYG**	**kanMX4**	**URA3**	**5**	**10**	**20**	**40**	**60**	**80**	**100**	**120**
A9-FZF1-d	−	+	−	+	−	−	−	−	−	−	−
A9-FZF1-u	−	−	+	+	+	+	+	+	+	+	−
A9-FZF1-e	−	−	+	+	+	+	+	+	+	+	−
A9-FZF1-u-s	+	−	−	+	+	+	−	−	−	−	−
A9	−	−	−	+	+	+	−	−	−	−	−

### qRT-PCR analysis

**Figure 2:**
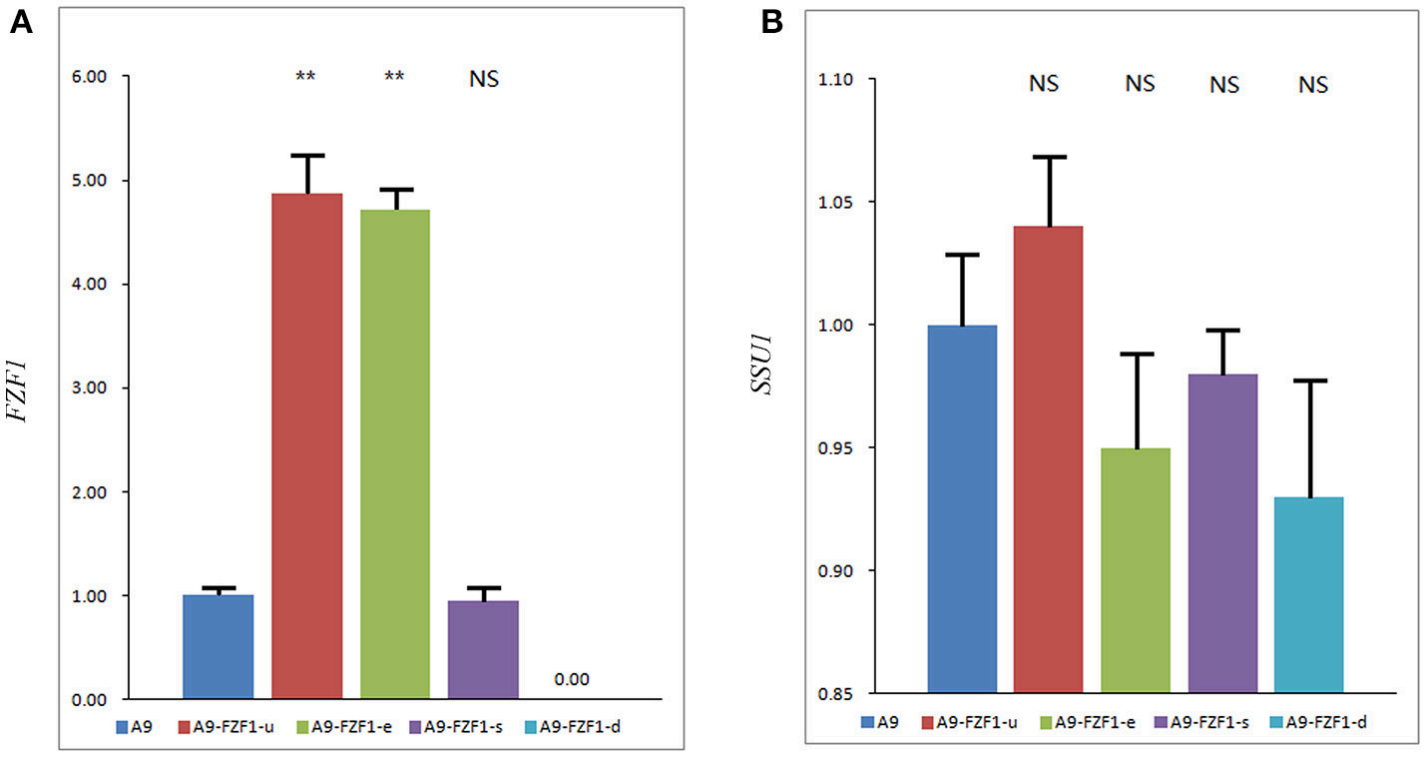
The expression level of the *FZF1* **(A)** and *SSU1* **(B)** genes in different strains. The expression levels of genes were assessed using a 2^-ΔΔC_T_^ method to determine the relative gene expression from qPCR data with *ACT1* as a housekeeping gene. Values are 2^-ΔΔC_T_^ Mean ± SE (*n* = 3). NS: *P* > 0.05, not significant, **P < 0.01, with one-way ANOVA. Note that different scales are used in the two figures. Total RNA failed to be extracted from A9-FZF1-u-d after treatment with sulfite, so the data of A9-FZF1-u-d were absent.

One transformant colony of each vector with the right genotypes and the expected PCR amplified bands was selected as representing that strain. qRT-PCR was used to quantify the average transcript concentration of the *FZF1* gene in these strains together with the starting strain A9. Figure 2A shows that A9-FZF1-u and A9-FZF1-e demonstrated increased transcription relative quantification (RQ) of *FZF1* by 4.87- and 4.72-fold, respectively, over that of A9, while transcription RQ in A9-FZF1-u-s was 0.96 times that of the starting strain (as shown in Figure 2). The increases in the two over-expressing strains were significant (*p* < 0.01), but there was no significant difference between A9 and A9-FZF1-u-s. These data show that both *FZF1* over-expressions were successful.

The expression levels of the *SSU1* gene's three transformed strains were also assessed using qRT-PCR (as shown in Figure 2B). All three strains showed similar levels of *SSU1* transcription, with no significant differences from the starting strain. These results show that over-expression of *FZF1* does not affect the expression level of the *SSU1* gene in *S. uvarum*, which is in direct contrast to the situation in *S. cerevisiae*.

### Determination of the growth curves of the recombinant strains

**Figure 3:**
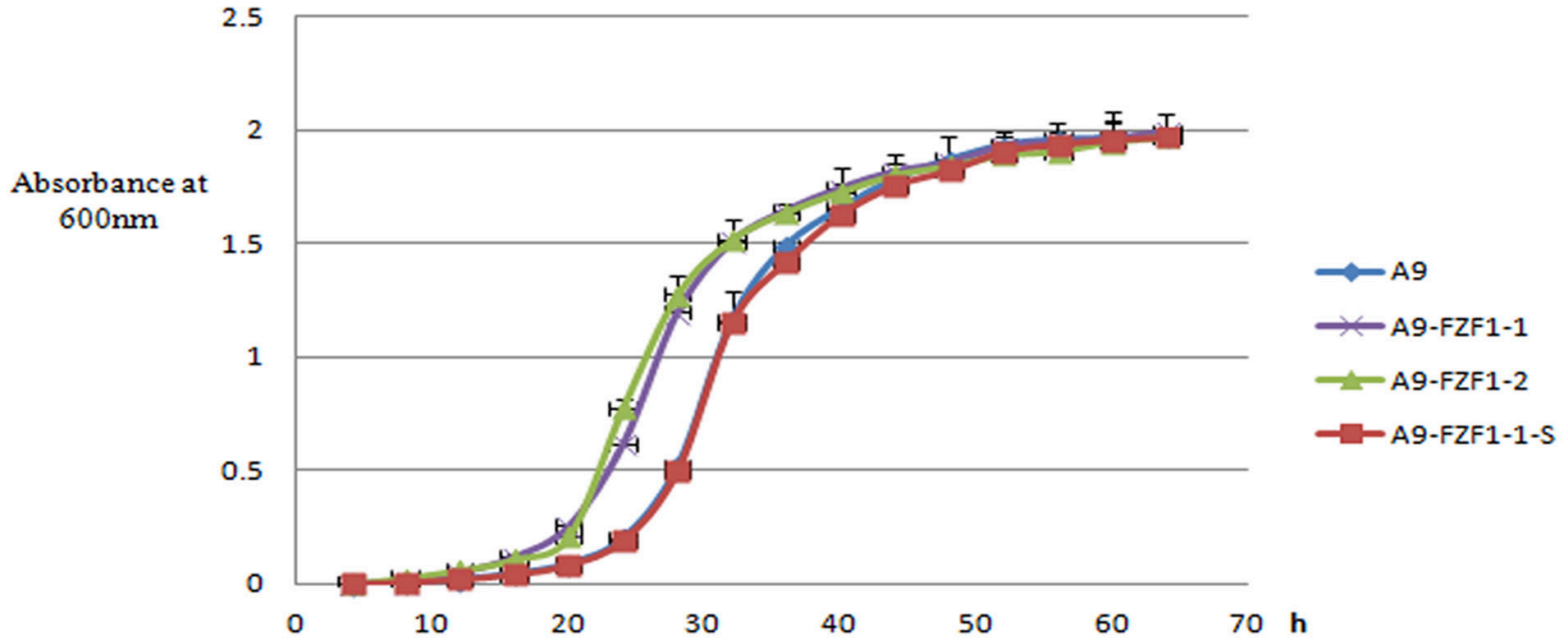
The growth curve of different strains. The data are plotted as the means and standard deviations of the triplicate values. The culture medium was fresh liquid YPD, and the data were collected every 4 h, with growth measured using light absorption at 600 nm.

The three representative strains, and the parental A9 strain, were cultured in YPD liquid medium in shake flasks. The lag phases (absorbance at 600 nm <0.10) of A9-FZF1-u-s and A9 were almost the same: around 20 h. Meanwhile, the lag phases of the *FZF1-*over-expressing strains were also similar to each other at around 12 h, but were significantly shorter than the other two strains (*p* < 0.00001). The average maximum growth rates for the triplicate values of A9, A9-FZF1-u, A9-FZF1-e, A9-FZF1-u-s, and A9-FZF1-u-d were 0.201 (±0.014), 0.220 (±0.013), 0.331 (±0.023), 0.209 (±0.027), and 0.196 (±0.007) h^−1^, respectively. There was no significant difference (*p* > 0.05) between the maximum growth rates of A9, A9-FZF1-u-s, and A9-FZF1-u-d. All of them were significantly smaller than that of A9-FZF1-u and A9-FZF1-e (*p* < 0.001), however. The average final titers for the triplicate values of these strains were all similar (1.975 (±0.009), 1.995 (±0.015), 1.969 (±0.028), 1.969 (±0.022), and 1.965 (±0.012) × 10^7^ cells/ml, respectively). The experimental results showed that the *FZF1-*over-expressing strains grew faster than the starting strain A9, and that A9-FZF1-u-s did almost the same as A9 (see Figure 3).

### Transcriptome analysis

RNA sequencing was used to quantify the transcripts in each of the four strains (A9, A9-FZF1-u, A9-FZF1-e, and A9-FZF1-u-s) in which RNA samples were extracted from colonies after treatment with 20 mM of sodium sulfite and 80 mM of succinic acid for 10 min. Total sequence reads varied from 27 to 40 million per strain, and all of the total clean reads ratio of these strains were very high (from 99.64% in A9-FZF1-u-s, to 99.94% in A9, see Table S1).

All 8490 unigene sequences were clustered using the clusters of orthologous groups (COG) function classification. There are 25 functional categories, such as RNA processing and modification, chromatin structure and dynamics, and energy production and conversion. The unigene sequences numbers within each cluster ranged from two to 1241, with an average number of 339.6 unigene sequences per group. There were 726 unigene sequences up-regulated in both A9-FZF1-e and A9-FZF1-u after subtracting vector-derived genes out of this total. Of these, 514 unigene sequences were also found to be up-regulated in A9-FZF1-u-s. Therefore only 212 unigene sequences were found to be up-regulated in *FZF1-*over-expressing strains. These unigene sequences represent candidates for altered regulation directed by the over-expression of *FZF1* in the two strains (see Table S2). There were 364 unigene sequences up-regulated in A9-FZF1-u-s, and 219 of them were also found to be up-regulated in A9-FZF1-e and A9-FZF1-u.

A total of 112 transcription factors (see Table S3) appeared in all four strains. The Log2 FPKM (expected number of fragments per kilobase of transcript sequence per million base pairs sequenced) values of the transcription factors ranged from -9.9658 in all strains to 11.5954 in A9, 9.8497 in A9-FZF1-u-s, 10.0773 in A9-FZF1-u, and 10.05140 in A9-FZF1-e. Results showed that the two over-expressing *FZF1* gene transformants have the most similar overall expression pattern of their transcription factors.

**Figure 4:**
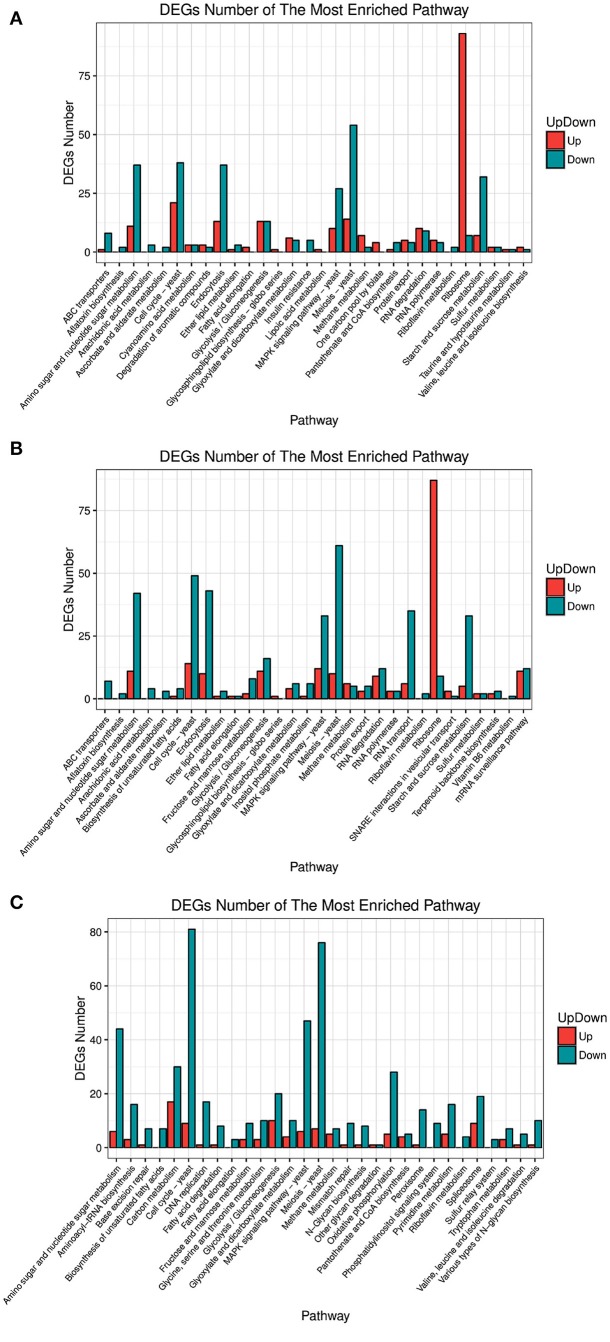
Pathway enrichment of different expression genes between *FZF1* transformed strains and A9 with Gene Ontology (GO) interpretation. **(A)** A9-FZF1-u vs. A9; **(B)** A9-FZF1-e vs. A9; **(C)** A9-FZF1-u-s vs. A9.

Analysis of the differentially expressed genes (DEGs) by function showed that the most enriched pathways differed between strains over-expressing different *FZF1* genes and the *FZF1*-RNAi fragment (see Figure 4). Among them, all the genes concerning a sulfur relay system were down-regulated in the A9-FZF1-u-s, but there were up-regulated and down-regulated genes in the transformed strains of the different *FZF1* genes. The gene numbers of categories, such as membrane transport and translation in over-expression strains, were increased compared to those in the silenced one, while the gene numbers of categories such as replication and repair, energy metabolism, lipid metabolism, amino acid metabolism, carbohydrate metabolism, and cell growth and death in over-expression strains decreased.

Table 3 shows the expression data for a selection of sulfur-related genes assessed for the ratio of expression (comparison of the average of the two overexpressed strains to the average of the A9 and silenced strain). Among them, *MET4* and *HAL4* were up-regulated in both FZF1*-*over-expressing strains, *PRPD* and *CYS3* were up-regulated only in A9-FZF1-u, and *ILV3, ELP3, NFS1*, and *CFD1* were significantly up-regulated only in A9-FZF1-e (see Table 3). Thus, *MET4* and *HAL4* are the leading candidates for involvement in sulfite tolerance via up-regulation of *FZF1*.

**Table 3:** List of up-regulated genes related to sulfite or sulfur metabolism in over-expressed strains compared with A9 and the silenced strain.

**GeneID**	**FPKM values of A9**	**FPKM values of A9-FZF1-u-s**	**FPKM values of A9-FZF1-u**	**FPKM values of A9-FZF1-e**	**The ratio of expression**	**Swiss-Prot-annotation**
Unigene4188_All	nd	Nd	1.17^a^	Nd	na	sp|Q12428|PRPD_YEAST
Unigene4262_All	nd	Nd	0.48^a^	Nd	Na	sp|P31373|CYS3_YEAST
Unigene2980_All	15.53	26.78	40.5^b^	39.47^b^	1.890097	sp|P32389|MET4_YEAST
Unigene3470_All	53.56	79.97	129.11^a^	82.84	1.587284	sp|P38068|GLRX7_YEAST
CL277.Contig2_All	18.85	23.66	25.85	39.9^a^	1.546695	sp|P39522|ILV3_YEAST
Unigene1290_All	209.51	242.02	327.38^a^^,^ ^b^	310.69^b^	1.413129	sp|P25333|HAL4_YEAST
Unigene3304_All	155.86	85.76	176.1	162.19	1.400091	sp|Q03103|ERO1_YEAST
Unigene3159_All	30.73	59.29	54.6	57.92	1.249944	sp|Q08960|TYW1_YEAST
Unigene1132_All	102.04	87.67	103.77	120.78^a^	1.183649	sp|P25374|NFS1_YEAST
Unigene1159_All	60.97	62.9	65.83	77.45^a^	1.156697	sp|Q02908|ELP3_YEAST
Unigene2588_All	24.02	41.17	39.98	34.4	1.140973	sp|Q6Q560|ISD11_YEAST
Unigene261_All	111.77	62.93	96.81	101.21	1.133486	sp|P07264|LEUC_YEAST
Unigene985_All	19.16	29.68	29.99	24.95	1.124898	sp|P39692|MET10_YEAST
Unigene2617_All	8.05	9.88	11.08	8.4	1.086447	sp|P32451|BIOB_YEAST
Unigene967_All	35.75	31.91	37.46	36.08	1.086905	sp|P40469|MET18_YEAST
Unigene884_All	18.16	21.73	18.48	23.63^a^	1.055653	sp|P40558|CFD1_YEAST
Unigene1107_All	5.93	9.73	9.32	7.21	1.055556	sp|P47170|IML1_YEAST
Unigene1708_All	129.61	193.05	182.75	151.22	1.035052	sp|P32582|CBS_YEAST

The ratio of expression was calculated by the average of the two overexpressed strains divided by the average of A9 and the silenced strain;

a the value was the biggest among four strains and Q < 0.01;

b *The values of both FZF1-over-expressing strains were significantly bigger than those of A9 and the silenced strain and Q < 0.01*.

## Discussion

In *S. cerevisiae*, the *FZF1* gene was found to be a positive regulator of *SSU1* and to be involved in sulfite tolerance. It was found that multi-copy *FZF1* genes could result in producing more efflux acting through Ssu1p and Met20p (Avram and Bakalinsky, 1996). Here, we have confirmed that over-expression of *FZF1* also confers sulfite tolerance in *S. uvarum*. We also found, however, that over-expressing of the *FZF1* gene in *S. uvarum* did not lead to the expression change of the *SSU1* gene, but that over-expressing and deletion of it led to a change of sulfite tolerance. It confirmed the previous findings that sulfite resistance in the *S. uvarum* isolates is linked to *FZF1*, but not to *SSU1*.

Although *GCV1*, as well as *SSU1*, was found to be the target gene of *FZF1* besides *SSU1* in *S. cerevisiae* in a previous study (Hu et al., 2007), there was no evidence to infer that *GCV1* was influenced by the over-expressing of *FZF1* in this study. Compared to the 203 target genes of *S. cerevisiae* in the YEASTRACT database (Teixeira et al., 2014), however, 212 unigene sequences were found to be up-regulated in both *FZF1-*over-expressing strains, but not in A9-FZF1-u-s, and this group of genes should include the candidates for the *FZF1* targets. The sulfur-related genes among these included *MET4* and *HAL4*, which showed a 50-80% increase in transcription in both *FZF1-*over-expressing strains compared to A9 and the silenced strain.

Previously, studies have revealed that *MET4* and *HAL4* are involved in the sulfur metabolism in *S. cerevisiae* (Fauchon et al., 2002; Lee et al., 2010; Gey et al., 2014). In this study, *MET4* and *HAL4* were significantly up-regulated in both FZF1-over-expressing strains, meaning that they could be involved in the sulfite metabolism in *S. uvarum*. The protein encoded by *MET4* is a leucine zipper, and once it assembles onto the promoters, Met4p can recruit other transcriptional coactivator complexes, including Mediator and Spt-Ada-Gcn5-Acetyltransferase (SAGA) (Leroy et al., 2006; Su et al., 2008). This might be one of the reasons why there were up to 112 transcription factors (see Table S3) that changed their expression levels in this study. Here, *MET4* and *HAL4* were first found to be positively regulated by *FZF1* in *S. uvarum*.

Around 15% of the nucleotide sequences are different between *FZF1-u* and *FZF1-e*, which leads to 21% of the coded protein sequences being different. The changes in protein sequences may lead to a change of protein structure and function, and in the expression of the other genes involved. Two genes, namely *PRPD* and *CYS3*, were up-regulated only in A9-FZF1-u, and *ILV3, ELP3, NFS1*, and *CFD1* were significantly up-regulated in A9-FZF1-e. These differences might be caused by the expression of different sequences of the *FZF1* genes. Regarding sulfur metabolism, however, these differential genes concerning sulfur metabolism could not cause a sulfite tolerance difference as there was no significant difference between A9-FZF1-u and A9-FZF1-e.

RNAi is an ancient mechanism present in plants, animals, and most fungi, and is considered to be a type of genetic immune system (Ghildiyal and Zamore, 2009; Malone and Hannon, 2009). In this study, however, the results showed that the RNAi of the *FZF1* gene had failed. As the alleles of *DCR1* and *AGO* play vital roles in the mechanism of RNAi (Moazed, 2009), we checked the whole genome of A9, which has been previously sequenced (Zhang et al., 2015), but no alleles of *DCR1* and *AGO* were found. The idea that RNAi had been lost during the evolution of budding yeasts (Moazed, 2009) also seems to have been confirmed in this study. Although the depression of *FZF1* with RNAi was not working, it was still good to use A9-FZF1-u-s as another control besides A9 for comparing with the *FZF1*-overexpression strains. Among those changed genes, the *SLM3, NCS6*, and *TCD2* genes, which might concern the sulfite metabolism, were all up-regulated in A9-FZF1-u-s, but the mechanism was unknown and should be studied in future research.

Several of the mechanisms and strain-dependent strategies used to obtain sulfite resistance can deeply influence wine quality (Nadai et al., 2016). Zinc finger proteins represent some superfamily of nucleic acid binding proteins in eukaryotes cell that take part in a variety of cellular activities, such as differentiation, development, and cell cycle. Here, we have provided direct evidence that the *FZF1* gene plays an important role in regulating the sulfite tolerance in *S. uvarum*, and suggest the involvement of *MET4* rather than *SSU1* in this process. Again, the results confirm the conclusion of previous studies that *FZF1* is required for sulfite tolerance in *S. uvarum*, but that *SSU1* is not linked to this trait. This is the first observation that the different genes of the sulfur metabolism network can be up-regulated or down-regulated when an *FZF1* gene of different origin is over-expressed in the cells of *S. uvarum*.

## Conclusion

In conclusion, our results confirmed that the *FZF1* gene is important in the sulfite resistance mechanism of *S. uvarum*. Over-expression of *FZF1* derived from the *S. uvarum* strain A9 did not increase sulfite tolerance compared to the over-expression of *FZF1* derived from *S. uvarum* strain ACY338. Meanwhile, the deletion of the *FZF1* gene led to a decrease in sulfite tolerance. Instead of promoting the expression of *SSU1*, the expression of *MET4* and *HAL4* was increased. The *FZF1* gene, therefore, plays a different role in *S. uvarum* compared to its role in *S. cerevisiae*.

## Author contributions

XZL analyzed the experimental data and drafted the manuscript. XZL, XPL, and ZZ conducted the study. MS and XS helped analyze the experimental data. CH and PX participated in the coordination of the study. HZ conceived of the study and contributed to writing the manuscript. All authors approved the final version of the manuscript.

### Conflict of interest statement

The authors declare that the research was conducted in the absence of any commercial or financial relationships that could be construed as a potential conflict of interest.
